# Targeting WNT5B and WNT10B in osteosarcoma

**DOI:** 10.18632/oncotarget.28617

**Published:** 2024-08-02

**Authors:** Gustavo A. Miranda-Carboni, Susan A. Krum

**Affiliations:** ^1^Department of Medicine, University of Tennessee Health Science Center, Memphis, TN 38163, USA; ^2^Center for Cancer Research, University of Tennessee Health Science Center, Memphis, TN 38163, USA; ^3^Department of Orthopaedic Surgery and Biomedical Engineering, University of Tennessee Health Science Center, Memphis, TN 38163, USA

**Keywords:** WNT5B, WNT10B, WNT signaling, osteosarcoma

## Abstract

WNT signaling regulates osteosarcoma proliferation. However, there is controversy in the field of osteosarcoma as to whether WNT signaling is pro- or anti-tumorigenic. WNT-targeting therapeutics, both activators and inhibitors, are compared. WNT5B, a β-catenin-independent ligand, and WNT10B, a β-catenin-dependent WNT ligand, are each expressed in osteosarcomas, but they are not expressed in the same tumors. Furthermore, WNT10B and WNT5B regulate different histological subtypes of osteosarcomas. Using WNT signaling modulators as therapeutics may depend on the WNT ligand and/or the activated signaling pathway.

## OSTEOSARCOMA

Osteosarcoma is a type of bone cancer that affects about 400 children and young adults each year in the United States [1]. Patients are treated with chemotherapy and surgery, as there currently are no targeted therapies. The 5-year survival for localized disease is 76%, but only 24% for metastatic osteosarcoma. Therefore, the biology of osteosarcoma is actively being investigated to identify novel therapeutics.

## WNT SIGNALING

**Figure 1:**
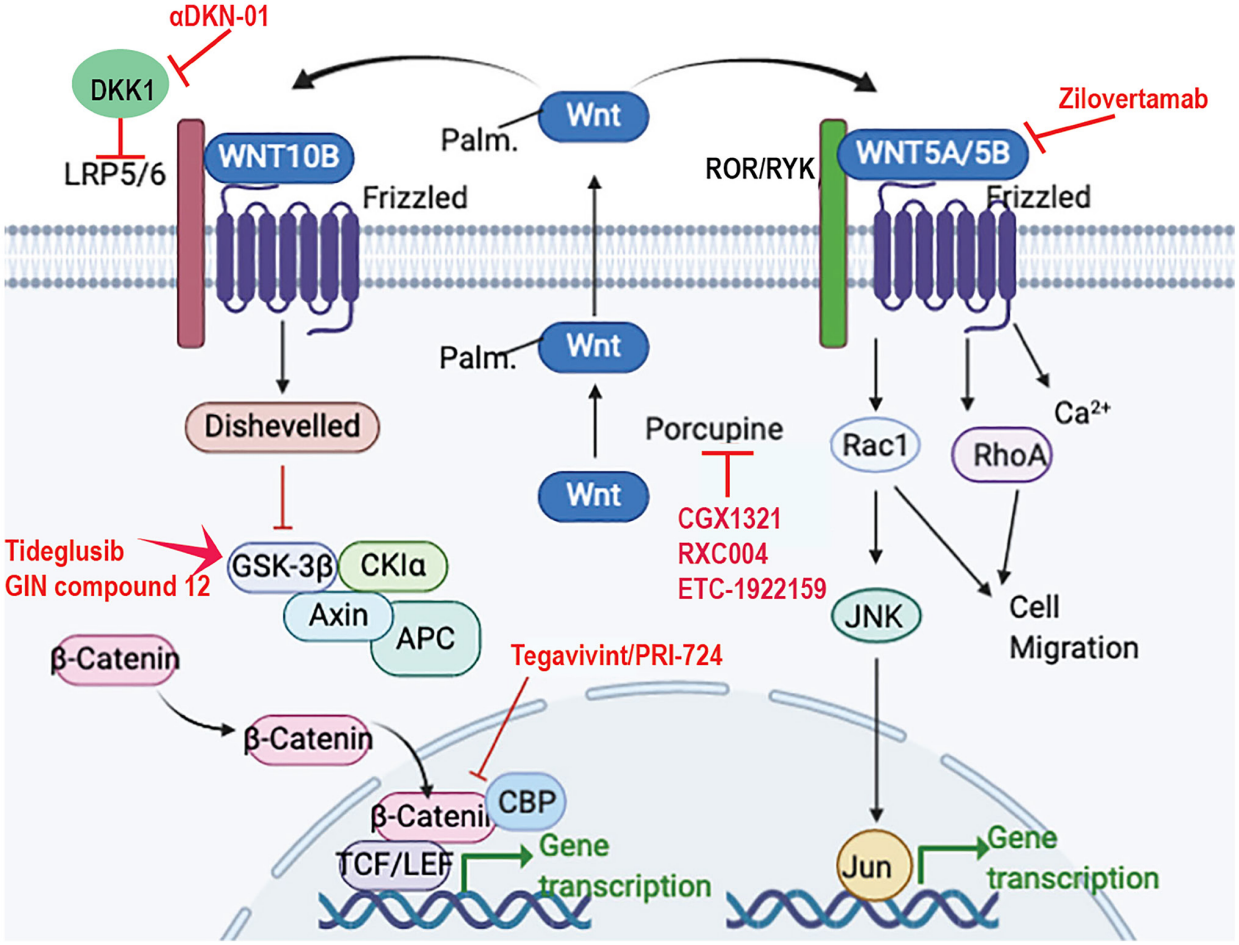
WNT signaling and proposed drug treatments for osteosarcoma. WNT ligands depend on porcupine-mediated palmitoylation (palm.) for secretion, which can be blocked by porcupine inhibitors such as CGX1321, RXC004, and ETC-1922159. Canonical WNT ligands, such as WNT10B bind to the receptors LRP5/6 and FZD to induce β-catenin stabilization and transcriptional activation. Tegavivint and PRI-724 inhibit β-catenin and CBP interaction preventing transcription. Tideglusib and GIN compound 12 activate GSK-3β leading to β-catenin stabilization and activation. αDKN-01 will block DKK1, thereby activating the canonical pathway. Zilovertamab is a monoclonal antibody to ROR1 to inhibit the non-canonical pathway induced by WNT5A and WNT5B. Created in https://www.biorender.com/.

Abnormal WNT signaling has been implicated in driving proliferation and/or stem cell growth in many cancer types, including osteosarcoma [2, 3]. Some WNT ligands, including -1, -3A, and -10B are often referred to as activators of β-catenin-dependent gene transcription (or “canonical WNT signaling”) (Figure 1). Canonical WNT ligands bind to the receptors LRP5/6 and a FZD coreceptor to initiate WNT signaling. The signaling output of the canonical WNT pathway is determined by the level of nuclear, activated β-catenin, which is under the strict control of the “destruction complex”. The core “destruction complex” is composed of AXIN, APC, and two constitutive active kinases (CK1α/δ and GSK3α/β), which associate with β-catenin and promote its polyubiquitination and proteasomal degradation by phosphorylating the degron motif of β-catenin. In the presence of a WNT ligand, the inactivated deconstruction complex releases β-catenin from proteasomal degradation, thereby causing cytoplasmic accumulation and nuclear translocation to control gene expression [4].

In contrast, β-catenin-independent (“non-canonical WNT signaling”) gene transcription-mediated WNT-ligands (e.g., -5A and -5B) transduce through ROR1, ROR2, and/or RYK receptors and a FZD co-receptor to activate RAC1/2 and/or RHOA kinases to downstream effectors such as p38 and JNK, among other pathways [5] (Figure 1). The β-catenin-independent pathways induce either calcium signaling or the Planar Cell Polarity (PCP) pathway. WNT/Ca^2+^ signaling leads to cellular migration and cytoskeletal changes and the activation of WNT/PCP signaling results in cell polarity, cell migration, and convergent extension.

## WNTs AND STEM CELLS

WNT signaling has been shown to regulate the self-renewal and differentiation of many types of stem cells [6]. Importantly for osteosarcoma, WNT signaling directs the differentiation and lineage specification of osteoblasts from mesenchymal stem cells [7]. WNT signaling also regulates cancer stem cells (reviewed in references [8, 9]), including in osteosarcoma [10].

## WNTs IN OSTEOSARCOMAS

Perkins, et al., analyzed RNA-sequencing of 107 osteosarcoma patient samples from the St. Jude PeCan Data Portal and 97 patient samples from the TARGET dataset for the expression of all 19 WNT ligands. The two most expressed WNT ligands in each dataset are WNT5B and WNT10B, composing ~36% of the total samples when combined [10]. WNTs 4, 5A, 7B, 11, and 16 are each expressed in a small percentage of tumors. In contrast, 26–33% of tumors do not express a WNT ligand.

There is a controversy in the field of osteosarcoma: is WNT signaling pro- or anti-tumorigenic [11]? Are WNT inhibitors or activators effective therapeutics? Several WNT/β-catenin pathway activators have been shown to inhibit osteosarcoma growth [12–14]. Dickkopf-1 (DKK1) is a canonical WNT signaling antagonist. It is a secreted glycoprotein that inhibits the LRP5/6 receptors (Figure 1). Treatment with an anti-DKK1 antibody, thus activating canonical WNT signaling, was shown to inhibit osteosarcoma tumor growth in mouse orthotopic models [12]. An anti-DKK1 antibody (αDKN-01) is in clinical trials for solid tumors (https://clinicaltrials.gov/ ID NCT04681248).

Tideglusib is a small-molecule inhibitor of GSK-3β, and, thus, activates WNT signaling by inhibiting the destruction complex to activate β-catenin nuclear translocation (Figure 1). Tideglusib was shown to reduce proliferation and induce apoptosis in MG63, U2OS, and 143B osteosarcoma cells *in vitro* and to decrease tumor growth of U2OS cells in mice *in vivo* [13]. A second GSK3β inhibitor (GIN), compound 12 from Lilly Research Laboratories, was also shown to inhibit the proliferation of MG63 and U2OS cells *in vitro* [14].

In contrast, several nuclear β-catenin inhibitors decrease osteosarcoma tumor growth in preclinical studies. Tegavivint and PRI-724 are two small molecules that inhibit the transcriptional activation activity of β-catenin (Figure 1), and both have been shown to decrease osteosarcoma proliferation and migration [15, 16]. Tegavivint is in clinical trials for solid tumors (ClinicalTrials.gov ID NCT04851119). A clinical study of PRI-724 in solid tumors was terminated due to low enrollment (NCT01302405).

## WNT5B IS EXPRESSED IN 20–39% OF OSTEOSARCOMAS

WNT5B is a non-canonical WNT ligand that is widely expressed in many tissues, both normal and pathogenic [17, 18]. In bone, WNT5B inhibits osteoblast differentiation and mineralization [19], while driving osteoclast differentiation [20].

In the osteosarcoma PeCan and TARGET datasets, *WNT5B* is expressed in 20 and 24% of tumors, respectively. It is surprising that *WNT5B* is the most expressed WNT in osteosarcoma because WNT5B had not been previously studied in osteosarcoma, until Perkins et al., 2023. High expression of *WNT5B* is correlated with a low probability of survival. Immunohistochemistry (IHC) on tumor microarrays (TMAs) demonstrated that WNT5B protein was scored at medium or high levels in 39% of the 80 samples, consistent with the mRNA expression from multiple datasets [10].

WNT5B expression is high in osteosarcoma stem cells and the addition of recombinant WNT5B to sarcospheres *in vitro* led to increased sphere size (an indication of cancer stem cells) and increased chemoresistance to methotrexate. Knockout of *WNT5B* or inhibition of WNT5B signaling with a pre-clinical antibody to its receptor ROR1 (similar to Zilovertamab) decreased the sphere size and chemoresistance to methotrexate. Mechanistically, WNT5B led to the upregulation of SOX2, a stem cell marker and transcription factor that maintains stemness [10]. Future studies should test the inhibition of ROR1 in WNT5B-expressing osteosarcomas *in vivo* using Zilovertamab or a similar drug [21].

## WNT10B IS EXPRESSED IN 16–18% OF OSTEOSARCOMAS

One of the key WNT ligands in normal bone is WNT10B [22, 23]. The role of WNT10B in bone *in vivo* has been analyzed using *Wnt10b* global knockout mice, which have a loss of progenitor mesenchymal stem cells (MSCs) and loss of bone mass [24]. *In vitro* addition of WNT10B increases osteoblastogenesis of mesenchymal precursors [25]. 16–18% of osteosarcoma primary tumors express *WNT10B* [10]. When *WNT10B* is highly expressed in osteosarcomas there is a correlation with worse survival outcomes [26]. The mechanism of WNT10B in osteosarcomas has not been elucidated.

## *WNT5B* AND *WNT10B* ARE NOT EXPRESSED IN THE SAME OSTEOSARCOMA TUMORS

**Figure 2:**
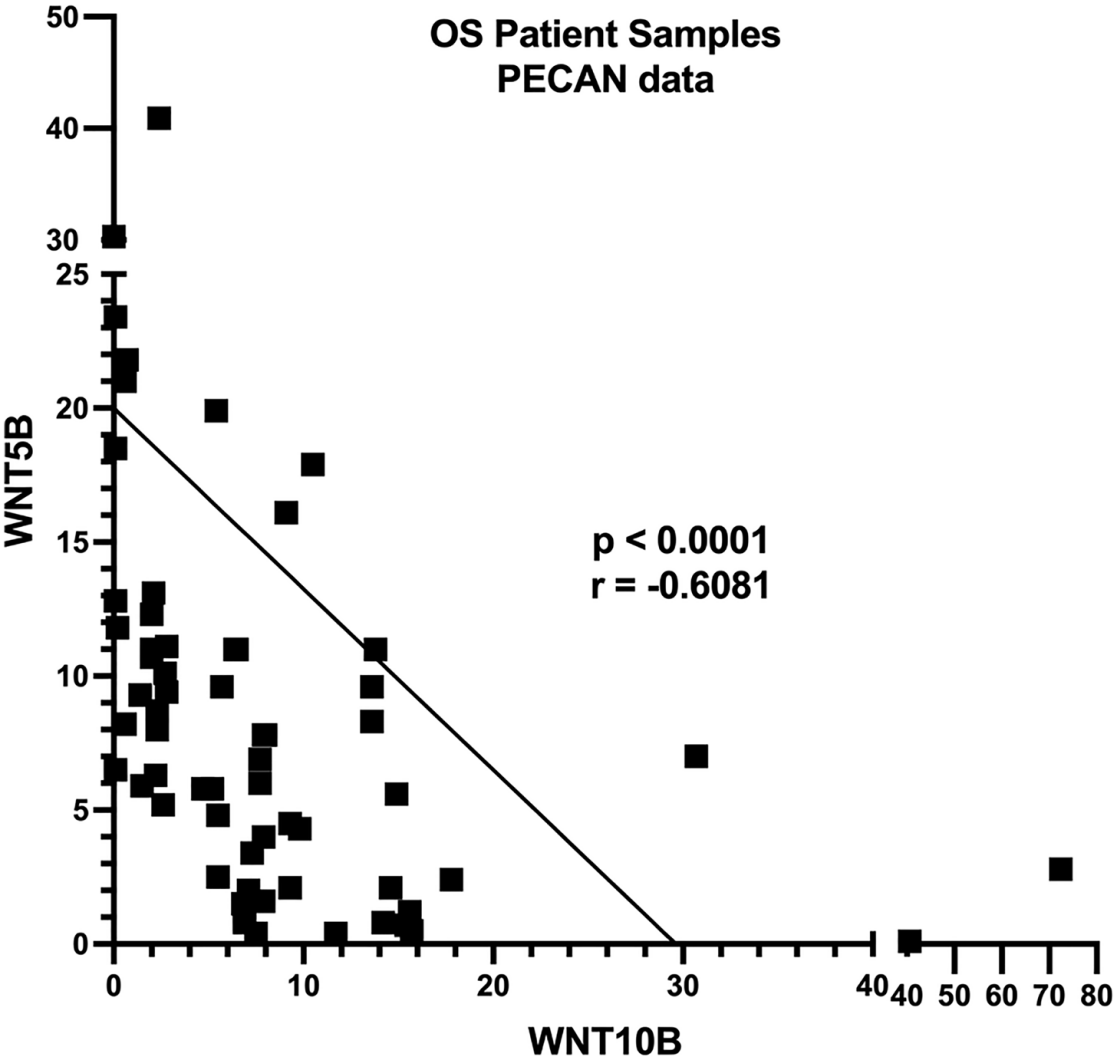
*WNT10B* and *WNT5B* are not expressed in the same tumors. Osteosarcomas in the St. Jude Children’s Hospital Pediatric Cancer (PeCan) Data Portal were analyzed for *WNT5B* and *WNT10B* expression. *p* < 0.0001.

Analysis of 107 samples from the PeCan data portal (*p* < 0.0001), 15 osteosarcoma PDX tumors from St. Jude Children’s Research Hospital (*p* < 0.05, data not shown), and 23 osteosarcoma tumors from GEO dataset GSE36004 [27] (*p* < 0.02, data not shown) revealed that *WNT5B* and *WNT10B* are not expressed in the same tumors (Figure 2). When either *WNT10B* or *WNT5B* are expressed, there is an inverse correlation in their expression. Therefore, a treatment against WNT10B or WNT5B signaling pathways will only work on a subset of cancers because they activate different pathways. A β-catenin nuclear inhibitor such as PRI-724 or tegavivint would theoretically not work on the osteosarcoma tumors that express *WNT5A* or *WNT5B* (~50% of tumors) or those that do not express a WNT ligand (~30% of tumors), and conversely, a ROR1 inhibitor would not work on osteosarcoma tumors that express *WNT10B* only.

## WNT10B AND WNT5B REGULATE DIFFERENT HISTOLOGICAL SUBTYPES OF OSTEOSARCOMAS

**Figure 3:**
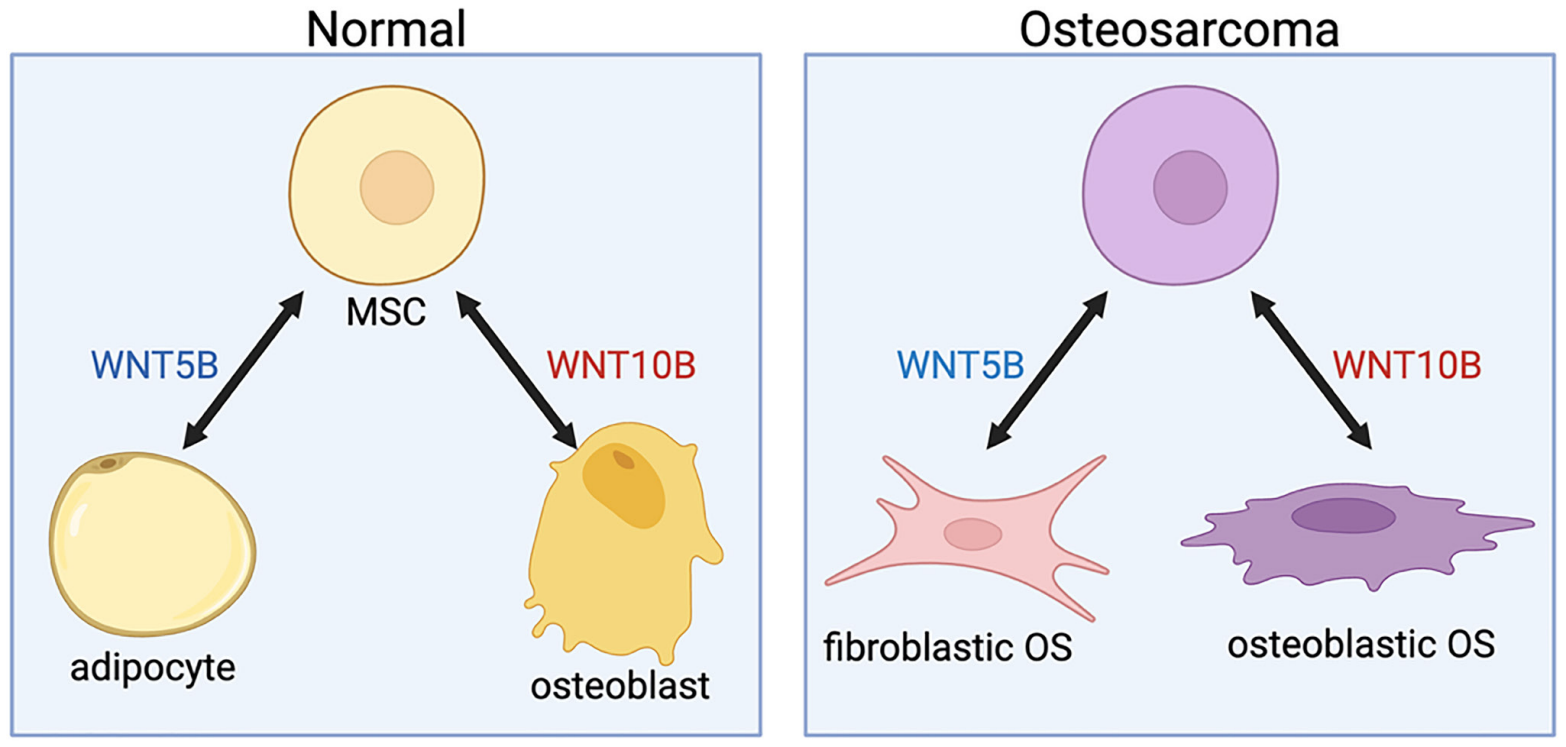
WNT5B and WNT10B in normal tissue vs. osteosarcomas. Osteoblasts and adipocytes differentiate from mesenchymal stem cells (MSCs). WNT10B increases osteoblast differentiation, while WNT5B induces adipocyte differentiation (left panel). Similarly, WNT10B correlates with osteoblastic osteosarcomas (OS), and WNT5B correlates with fibroblastic OS (right panel). Created in https://www.biorender.com/.

Osteosarcomas can be categorized based on their histological subtypes, including osteoblastic, fibroblastic, chondroblastic, and others. Perkins, et al. showed that *WNT5B* is more highly expressed in the fibroblastic subset of osteosarcoma [10], in comparison to the osteoblastic subset. In comparison, WNT10B regulates osteoblastic differentiation and is more expressed in the osteoblastic subtype of osteosarcoma (Figure 3). We identified osteoblast differentiation genes (*SP7* (osterix), *ALPL*, *BMP4*, and *PHOSPHO1*) in RNA-sequencing of osteosarcoma patient tumors and *WNT10B* correlated positively with these genes, while these genes inversely correlated with *WNT5B* (Table 1). Furthermore, we have shown that WNT5B inhibits normal osteoblast differentiation [19] and it inversely correlates with osteoblast differentiation genes in osteosarcomas (Table 1 and Figure 3). Instead, *WNT5B* correlates with *CD44*, a stem cell marker [28], and fibronectin (*FN1*), a fibroblast marker [29].

**Table 1:** Correlation of osteoblast genes with *WNT10B* and *WNT5B*

	WNT10B		WNT5B	
	R	*p*-value	R	*p*-value
CD44	−0.396	4.18E-06	0.629	2.9E-12
Fibronectin (FN1)	−0.337	1.05E-04	0.560	2.21E-9
Osterix (SP7)	0.512	5.33E-04	−0.531	2.05E-07
ALPL	0.448	1.94E-05	−0.594	2.58E-09
BMP4	0.474	5.29E-06	−0.252	0.021
PHOSPHO1	0.584	5.66E-09	−0.468	6.99E-06

Data from R2: Genomics Analysis and Visualization Platform. [30].

## CONCLUSIONS

Recent work has highlighted the importance of WNT5B signaling in osteosarcomas [10], reigniting the interest in targeting WNT signaling in osteosarcomas. WNT5B signaling could be inhibited with a ROR1 antibody such as Zilovertamab. There are currently no WNT modulators in specifically osteosarcoma trials, but there are a few trials targeting WNT in solid tumors (FOG-001, E7386, SM08502, and ST316 (β-catenin inhibitors), CGX1321, RXC004 and ETC-1922159 (porcupine inhibitors), and 9-ING-41 (a GSK-3β inhibitor)) [31]. Targeting WNT signaling in a few clinical trials for other cancer types has not been shown to be successful and this could be because they are targeting all tumors without determining the activating pathway. In addition, canonical WNT inhibitors lead to an increase in bone fractures [32], but a non-canonical inhibitor would not. As there is controversy over whether we should use WNT activators or WNT inhibitors to treat osteosarcoma, we hypothesize that it depends on whether the canonical or non-canonical pathways are activated, and this remains to be formally tested.
